# Evaluation of the effect of adjunctive diode laser application on peri-implant crevicular fluid biomarker levels: a randomized controlled trial

**DOI:** 10.1007/s00784-024-05855-4

**Published:** 2024-07-31

**Authors:** Nazan Ece Erduran, Guliz N. Guncu, Abdullah C. Akman, Buket Acar, Asli Pinar, Erdem Karabulut, Rahime M. Nohutcu

**Affiliations:** 1 Department of Periodontology, Faculty of Dentistry, Hacettepe University, Sihhiye, Ankara, TR-06230 Turkey; 2 Department of Biochemistry, Faculty of Medicine, Hacettepe University, Ankara, Turkey; 3 Department of Biostatistics, Faculty of Medicine, Hacettepe University, Ankara, Turkey

**Keywords:** Peri-implantitis, Peri-implant crevicular fluid, Diode laser, Biomarker

## Abstract

**Objectives:**

To assess both the clinical and immunological effectiveness of diode laser therapy when used as an adjunct to non-surgical mechanical therapy in managing peri-implantitis.

**Materials and methods:**

A cohort of 27 participants, comprising 21 females and 6 males, agreed to take part in this investigation. 37 dental implants with peri-implantitis diagnosis were randomly allocated to either the laser group (*n* = 19) or the control group (*n* = 18). Evaluation of peri-implant clinical parameters and collection peri-implant crevicular fluid (PICF) samples occurred at baseline, as well as at 3 and 6-month follow-up intervals. The level of various biomarkers (TWEAK, IL-1β, sclerostin, IL-17, RANKL, OPG and IL-10) within the PICF were quantified using enzyme-linked immunosorbent assay.

**Results:**

Significant time-dependent decreases in clinical and biochemical parameters were detected in both groups compared to the baseline. There were marked differences between the groups in terms of periodontal parameters, except probing depth, and IL-1β, IL-17, sclerostin levels in PICF at 3rd month follow-up. However, no statistically significant difference was detected at 6th month.

**Conclusions:**

Diode laser seems to be a reliable tool as an adjunct for supporting the nonsurgical mechanical treatment during the early stages of peri-implantitis. Furthermore, the findings suggest that IL-17, sclerostin and IL-1β may serve as promising biomarkers for assessing efficacy of peri-implantitis treatment.

**Clinical relevance:**

Based on these outcomes, clinicians may consider the application of adjunctive use of diode laser to non-surgical peri-implantitis treatment to achieve better clinical and immunological improvements than nonsurgical peri-implantitis therapy alone in just early healing period. However, it should be noted that there was no difference between the two methods in the long term.

## Introduction

Currently, dental implants have been accepted as reliable treatment options in case of missing teeth. However, peri-implant mucositis [43% (95%CI: 32–54)] and peri-implantitis [22% (95%CI: 14–30)] are developed as a result of biologic complication in most of the cases and impaired dental implant survival and success [1–3]. Peri-implant mucositis presents as inflammation within the soft tissue, while peri-implantitis is a condition also characterized by progressive bone loss in supporting peri-implant area [4]. Unmanaged peri-implant mucositis may proceed to peri-implantitis and untreated peri-implantitis may lead to loss of dental implants. Due to the high prevalence rates of peri-implantitis, nowadays, a comprehensive understanding of the pathogenesis of peri-implant diseases, their prevention, and effective management should be the most important part of dental implant treatment [2].

In literature, there were several therapeutic approaches suggestions for management of peri-implantitis, including nonsurgical, resective or regenerative surgical, and combined treatments with the use of anti-infective agents, laser application and air-abrasives [5, 6]. Currently, it’s still uncertain which interventions yield the highest efficiency in managing of peri-implant diseases [7]. Nonsurgical treatment procedures showed limited predictability in peri-implantitis treatment [8–12]. On the other hand, surgical non–regenerative procedures for peri-implantitis treatment may lead to short-term reduction in inflammation, but appears to be less effective over the long-term perspective [5, 13] and long-term data on outcomes after surgical treatment show greater improvement in alveolar bone levels [12, 14].

It’s clear that peri-implantitis treatment should definitely provide infection control by removing tissue deposits from the implant surface regardless of the applied treatment procedure either surgical or non-surgical [14, 15]. However, non-surgical or surgical approaches were reported to be not efficient in decontamination of the exposed dental implant surfaces in peri-implantitis treatment and supporting traditional approaches with adjunctive methods such as air-abrasives and lasers were highly suggested [16].

Lasers with various wavelengths (Er: YAG, diode lasers and CO_2_) have been proposed as adjuncts to non-surgical peri implant disease treatment to enhance outcomes [7]. In a comprehensive evidence review in 2018, a presence of an evidence showing controversial clinical benefits of non-surgical treatment of peri-implantitis by adjunct laser treatment in short term evaluations was reported [17]. Moreover, evidence is equivocal with regard to treatment outcomes for adjuvant application of especially diode lasers in nonsurgical peri-implantitis therapy. While positive outcomes in clinical peri-implant parameters were reported in some studies [18–20], no additional benefits were also obtained as diode laser groups resulted in comparable outcomes to the control groups in other studies [21–23]. Peri-implant crevicular fluid (PICF), found within the peri-implant sulcus, harbors significant biological constituents suitable for diagnostic and monitoring applications [24]. Indicating biomarkers in peri-implant crevicular fluid (PICF) that could detect response to peri-implant therapy and evaluating the host response profile around dental implants before and after performing peri-implantitis treatment will provide comprehensive information about the success of suggested peri implant treatment approaches [25, 26].

In a prior investigation conducted by our research team, levels of TWEAK, OPG and sclerostin were assessed in both PICF and gingival crevicular fluid (GCF) under diseased and healthy conditions. Inflammatory conditions were associated with notably elevated amounts of RANKL, sclerostin, TWEAK, and OPG [24]. Moreover, in another current study, levels of GCF IL-17, sclerostin and TWEAK levels have been proposed as valuable biomarkers for monitoring the response to periodontal therapy including scaling root planning and diode laser [27].

In the literature, there is inconsistency in the findings regarding the efficacy of using diode laser as an adjunctive treatment. The assessment of both clinical alterations and biomarker levels linked with the pathogenesis of peri-implantitis before and after the treatment may solve this discrepancy. Therefore, the primary objective of this randomized controlled clinical trial was to assess the clinical and immunological effectiveness of utilizing a diode laser (940 nm) in conjunction with non-surgical mechanical interventions in management of peri-implantitis.

## Materials and methods

### Study design, population and randomization

**Fig. 1:**
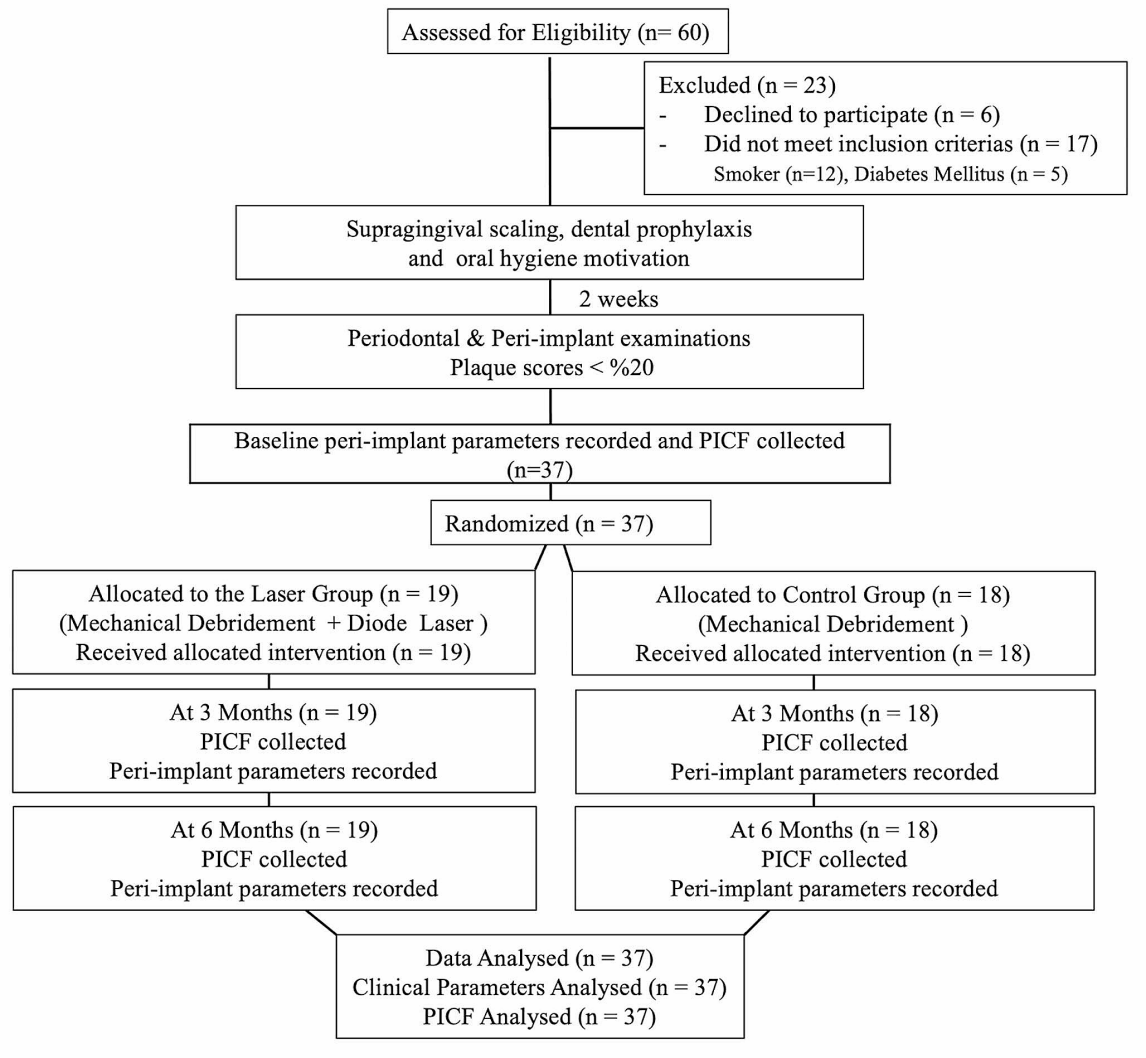
Flow-chart of the study

The schematic design of the present study is summarized in Fig. 1. It was planned as a prospective randomized, controlled, double-masked, clinical trial employing a parallel design of six month duration. A total of 27 individuals, systemically healthy and non-smokers, meeting the criteria of having at least one implant in function for a minimum of 6 months, aged 22–72 years (21 females and 6 males; mean age: 55.08 ± 10.57 years) were recruited from the Periodontology department, during the period from January 2020 to February 2022. This trial adheres to the principles of the Declaration of Helsinki for studies involving human subjects. The current protocol obtained ethical approval (Institutional Review Board of Hacettepe − 2019/ 11–32, KA-19059; 68869993-511.06-E.109597). Prior to enrollment, each volunteer was carefully briefed on the treatment procedure, potential risks and benefits and informed consent was obtained (ClinicalTrials.gov registration number: NCT05201443).

Inclusion criteria for participants were as follows: (a) individuals aged 18 or above; (b) absence of systemic chronic disease or medication known to influence periodontal health; (c) presence of at least one dental implant in functioning for a minimum of 6 months with a diagnosis of peri-implantitis without any keratinized tissue deficiency. Peri-implantitis diagnosis was based on the 2017 classification [28]. 

The applied exclusion criteria were as follows: (a) pregnancy or lactation; (b) receipt of any periodontal – peri-implant treatment within the preceding 6-months; (c) administration of antibiotics, probiotics or NSAIDs therapy in the preceding 6 months; (d) former or current smokers.

Two weeks before the beginning of this randomized - controlled clinical trial supra-gingival scaling and dental prophylaxis procedures were performed in a single visit with detailed oral hygiene educations. After 2 weeks peri-implant parameters were re-evaluated and individuals exhibiting full-mouth plaque scores of less than 20% were enlisted to the study.

Dental implants diagnosed with peri-implantitis, according to consensus report of workgroup 4 of the 2017 World Workshop on the Classification of Periodontal and Peri-Implant Diseases [4], were randomly allocated to the laser (L) and control (C) groups: L group included 19 implants (aged 46 to 72 years, mean age 56.88 ± 7.87) and C group included 18 implants (aged 22 to 68 years, mean age 56.36 ± 12.59). Assignment to the study groups was carried out according to a randomization code prepared on the computer beforehand. Concealed allocation was ensured through sealed, opaque envelopes containing control or laser assignments prepared in advance. To maintain blinding throughout the clinical study, the details of the groups and randomization code were known by one of the researchers (ACA) until analysis. The opaque envelope containing the code of the treatment, was unveiled after non-surgical treatment with a titanium curette (Titanium Implant Scaler, Hu-Friedy, Chicago, IL) administered to all dental implants to ensure impartiality.

### Clinical measurements and calibration

Clinical parameters including probing depth (PD), gingival recession (GR), gingival bleeding time index (GBTI) [29] plaque index (PI) [30] and gingival index (GI) [30] were assessed at baseline, 3 and, 6 months post-treatment, at four sites per dental implant using a periodontal probe (Michigan O Color-Coded Probe, Hu-Friedy, Chicago, IL). An experienced clinician (GNG) blinded to the study groups were conducted all clinical examinations. Before and during study period, intra-examiner calibration was obtained by assessing PD and GR in duplicate, with a degree of agreement within ± 1 mm higher than 85% at both tests.

### Peri-implant treatment

Following local anesthesia (Ultracain, Articain Hydroclorure, 20 mg/mL, Aventis Farma, Istanbul, Turkey), non-surgical mechanical treatment was carried out around each dental implant for 10 min with titanium curettes (Titanium Implant Scaler, Hu-Friedy, Chicago, IL) for removal of hard deposits. Subsequently, the inflamed peri-implant soft-tissue wall of the pocket was curetted with stainless steel curettes (Hu-Friedy, Chicago, IL). Eventually, the implant sulcus was irrigated with sterile saline solution. At this point of treatment in the L group, adjunctive diode laser (Epic, Biolase, Irvine, CA) therapy was applied in continuous phase at 940 nm wavelength, 0.80 W power, and 0.80 J/s energy level using an optic fiber tip with a diameter of 300 μm placed to the most apical part of the inner peri-implant pocket as parallel to the dental implant surface. The tip of the laser was systematically moved as apico-coronally and mesio-distally slowly and cleaned regularly with sterile gauze during the treatment for checking formation of blood coagulation. In C group, adjunctive diode laser tip was also applied into the peri-implant pockets as a non-activated way. All treatments were administered by the same experienced clinician (NEE).

### PICF sampling

PICF samples were collected at baseline, 3 and, 6 months post-treatments [31] from four sites per dental implant (mesiobuccal, mid-buccal, distobuccal, and mid-palatinal/lingual regions) using sterile paper strips Periopaper, OraFlow, Amityville, NY, USA). Following isolation of implant sites, supragingival plaque removal, and insertion of paper strips into the 1 mm depth at the entrance of gingival sulcus regardless of the PD for 30 s; samples were immediately transferred to a previously calibrated device (Periotron 8000, OraFlow, Amityville, NY) for volume measurement and values in terms of microliters were determined through a software program (MLCONVERT.EXE, Oraflow, Hewlett, NY). Subsequently, paper strips from each dental implant were put in a single Eppendorf tube and stored at -82 °C until ELISA analysis.

### Quantification of biomarkers in PICF

After the paper strips in Eppendorf tubes were kept at room temperature for at least 30 min, the content of paper strips was extracted by adding 800 µL of sterilized PBS (Phosphate Buffered Saline) and by vortexing. Following this step, the samples were centrifuged at 4 °C 10,000 rpm for 15 min. The levels of biomarkers in PICF samples were quantified using ELISA kits (USCN Life Science Kit, Cloud-Clone Corp., Wuhan, Hubei). The minimum detection level or lower level of detection (LLD) values for ELISA kits were less than as follows: For IL-1β 6.1 pg/mL; for IL-10 2.8 pg/mL; for IL-17 5.5 pg/mL; for sclerostin 0.131 ng/mL; for TWEAK 6.0pg/mL; for RANKL 1.27 pg/mL; for OPG 0.059 ng/mL. A standard curve was created according to the absorbance values ​​of standard microwells obtained from the spectrophotometer.

### Statistical analyses

A sample size of 18 dental implants per group was determined to achieve 85% power to detect a 1-mm difference in PD and GBTI between the laser and the control groups with an alpha value of 0.05. Statistical analyses were performed using SPSS software. The Kolmogorov-Smirnov and Shapiro-Wilk tests were employed to assess normal distribution for all parameters. Chi-square test was used to determine the differences between groups in terms of sex as a categorical variable. Mann-Whitney U Test was utilized to compare the differences among the groups, and within-group differences were evaluated with Related Samples Friedman’s ANOVA Test. Correlations were defined by Pearson and Spearman coefficient. All statistical analyzes were performed with the SPSS software (SPSS v-23, IBM Corp, NY, USA). Statistical significance was set as *p* < 0.05.

## Results

In the present study, a total of 60 dental implants were assessed for eligibility. 23 of these 60 dental implants were excluded since they did not meet the inclusion criteria and declined to participate. Subsequently, the planned treatments were performed on a total of 37 dental implants diagnosed with peri-implantitis and six-month follow-up periods were completed (Fig. 1). 15 patients (mean age: 56.88 ± 7.87) with 19 implants in the laser group, and 12 patients (mean age: 56.36 ± 12.59) with 18 implants in the control group were recruited. There was no significant difference between the groups in terms of sex and age (*p* > 0.05) (Table 1).

**Table 1:** Distribution of demographic variables in the laser and control groups

Demographic Variables	Laser Group (*N* = 19 Dental Implants)	Control Group (*N* = 18 Dental Implants)	*p*
**Age** mean ± SD (min-max)	56.88 ± 7.87 (46–72)	56.36 ± 12.59 (22–68)	0.318
**Sex (F/M)**	11/ 4	10/ 2	0.225

F: Female, M: Male, SD: standard deviation

Table 2 summarizes the values and statistical comparison of the peri-implant indices of the treated dental implants during the study period. Since peri-implant indices (PD, GI, PI, GBTI) did not differ between the groups at baseline assessment (*p* > 0.05), the laser and control groups were considered as comparable in terms of baseline periodontal status. While significant decreases were observed in periodontal parameters (PD, GI, PI, GBTI, PICF volume) in the post-treatment 3rd and 6th months compared to baseline values (*p* < 0.05), no significant difference was found between the 3rd and 6th months after treatment within the both groups; except for the PICF volume, which showed a significant decrease in the 6th month compared to the 3rd month in the two groups (*p* < 0.05). Although baseline levels did not differ between the groups, GI, PI and GBTI values were significantly lower in the laser group than in the conventional treatment group at the 3rd month after treatment (*p* < 0.05). However, these significant differences between the groups were not observed at the post-treatment 6th month (*p* > 0.05). The amount of PICF and PD did not display any significant difference between the groups at any follow-up period, as well as baseline assessment (Table 2).

**Table 2:** Comparisons of clinical measurements averages of dental implants during evaluation period

Periodontal Variables	Laser Group (*N* = 19) mean ± SD (min-max)				Control Group (*N* = 18) mean ± SD (min-max)				Laser Group vs. Control Group (*p* values)^2^		
	Baseline	3 Months	6 Months	*P* ^1^	Baseline	3 Months	6 Months	*P* ^1^	Baseline	3 Months	6 Months
PD (mm)	4.95 ± 0.88 (3.75–6.75)	3.58 ± 0.82^a^ (2–5)	2.83 ± 0.53^b^ (2-3.75)	**< 0.0001** ^*^	4.79 ± 1.13 (3.50–7.25)	3.13 ± 0.82^d^ (1.75–4.75)	3.11 ± 0.95^e^ (1-4.5)	**< 0.0001** ^*^	0.425	0.061	0.159
GI	2.01 ± 0.73 (1–3)	0.29 ± 0.44^a^ (0–1)	0.09 ± 0.25^b^ (0–1)	**< 0.0001** ^*^	1.96 ± 0.32 (1.25–2.75)	1.04 ± 0.78^d^ (0–2)	0.29 ± 0.49^e^ (0-1.5)	**< 0.0001** ^*^	0.730	**0.002** ^*^	0.313
PI	1.67 ± 0.66 (0–3)	0.26 ± 0.41^a^ (0–1)	0.07 ± 0.16 ^b^ (0-0.5)	**< 0.0001** ^*^	1.68 ± 0.59 (0-2.5)	0.71 ± 0.60^d^ (0-1.75)	0.31 ± 0.49^e^ (0-1.75)	**< 0.0001** ^*^	0.916	**0.013** ^*^	0.169
GBTI	2.80 ± 0.79 (1–4)	0.32 ± 0.49^a^ (0–1.5)	0.12 ± 0.32^b^ (0–1)	**< 0.0001** ^*^	2.44 ± 0.72 (0.75 − 3.75	1.13 ± 0.79^d^ (0–2.25)	0.36 ± 0.50^e^ (0–.,75)	**< 0.0001** ^*^	0.189	**0.002** ^*^	0.86
PICF (µl)	1.31 ± 0.64 (0.51–2.4)	0.70 ± 0.36^a, c^ (0.17–1.73)	0.58 ± 0.29^b^ (0.15–1.18)	**< 0.0001** ^*^	2.03 ± 0.34 (1.14–2.4)	1.36 ± 0.58^d, f^ (0.27–2.4)	0.97 ± 0.50^e^ (0.21–1.87)	**< 0.0001** ^*^	0.217	0.431	0.195

PD: Probing Depth; GI: Gingival Index; PI: Plaque Index; GBTI: Gingival Bleeding Time Index, PICF: in peri-implant crevicular fluid

p^1^: Related Samples Friedman’s Two-Way Analysis of Variance by Ranks

p^2^: Mann Whitney U Test

^a^: Statistically significant difference between baseline and 3 months in the laser group

^b^: Statistically significant difference between baseline and 6 months in the laser group

^c^: Statistically significant difference between 3 months and 6 months in the laser group

^d^: Statistically significant difference between baseline and 3 months in the control group

^e^: Statistically significant difference between baseline and 6 months in the control group

^f^: Statistically significant difference between 3 months and 6 months in the control group

When the periodontal indices of the areas with the deepest (D) peri-implant pocket measurement were evaluated within the groups, significant decreases were observed in D-PD, D-GI, D-GBTI and D-PI levels at the 3rd and 6th month follow-up periods compared to the baseline measurements (*p* < 0.05). However, there was no significant difference between the 3rd and 6th month assessments in both groups for the values of D-PD, D-GI, D-GBTI and D-PI (*p* > 0.05). While there were no differences in D-PD and D-GI between the groups at any assessment time, D-GBTI (*p* = 0.003) and D-PI (*p* = 0.007) were markedly lower in the laser-treated group than in the control group at the 3-month follow-up. (Table 3).

**Table 3:** Comparisons of deepest peri-implant pocket measurements in the treated areas of each dental implant during evaluation period and comparison of the gingival index (D-GI), plaque index (D-PI) and gingival bleeding time index (D-DKZI) values ​​in these regions over time

	D-PD				D-GI				D-GBTI				D-PI			
	mean ± SD (min-max)				mean ± SD (min-max)				mean ± SD (min-max)				mean ± SD (min-max)			
	Baseline	3 Months	6 Months	*p* ^1^	Baseline	3 Months	6 Months	*p* ^1^	Baseline	3 Months	6 Months	*p* ^1^	Baseline	3 Months	6 Months	*p* ^1^
**Laser Group** **(** *N* **= 19)**	6.63 ± 0.68 (6–8)	4.32 ± 0.95^a^ (2–5)	3.21 ± 0.92^b^ (2–5)	**< 0.0001** ^*^	2.11 ± 0.74 (1–3)	0.42 ± 0.61^a^ (0–2)	0.16 ± 0.38^b^ (0–1)	**< 0.0001** ^*^	2.95 ± 0.71 (2–4)	0.47 ± 0.77^a^ (0–3)	0.16 ± 0.37^b^ (0–1)	**< 0.0001** ^*^	1.74 ± 0.65 (0–3)	0.26 ± 0.45^a^ (0–1)	0.11 ± 0.32^b^ (0–1)	**< 0.0001** ^*^
**Control Group** **(** *N* **= 18)**	6.39 ± 0.85 (5–8)	3.83 ± 0.99^d^ (2–5)	3.39 ± 1.04^e^ (1–5)	**< 0.0001** ^*^	2.06 ± 0.24 (2–3)	1 ± 0.97^d^ (0–2)	0.39 ± 0.70^e^ (0–2)	**< 0.0001** ^*^	2.72 ± 0.67 (1–4)	1.5 ± 1.04^d^ (0–3)	0.72 ± 0.75^e^ (0–2)	**< 0.0001** ^*^	1.78 ± 0.88 (0–3)	1.06 ± 0.87^d^ (0–2)	0.50 ± 0.71^e^ (0–2)	**< 0.0001** ^*^
**p** ^**2**^	0.271	0.134	0.518		0.753	0.098	0.480		0.480	**0.003** ^*^	0.026		0.775	**0.007** ^*^	0.126	

p^1^: Related Samples Friedman’s Two-Way Analysis of Variance by Ranks

p^2^: Mann Whitney U Test

^a^: Statistically significant difference between baseline and 3 months in the laser group

^b^: Statistically significant difference between baseline and 6 months in the laser group

^d^: Statistically significant difference between baseline and 3 months in the control group

^e^: Statistically significant difference between baseline and 6 months in the control group

Table 4 summarizes the values and statistical comparison of the biochemical parameters during the study period. As shown in Table 4, PICF levels of IL-17, IL-1β and sclerostin were significantly lower in the laser-treated group compared to the control group at the post-operative 3rd month. There was no significant difference between the groups in terms of PICF cytokine levels at baseline and at the 6th month follow-up. PICF IL-17, IL-1β and TWEAK levels showed significant decreases in all sampling periods in both groups, except for the IL-1β PICF level in the control group between 3rd and 6th months values. The reduction in PICF IL-10 level from baseline to 6 months post-treatment were statistically significant in both groups (*p* = 0.006 for the laser group *p* < 0.0001 for the control group). Moreover, the amount of IL-10 in the control group was significantly lower at the 3rd post-op month than the baseline level (*p* < 0.0001). In the laser-treated group, sclerostin displayed a significant decrease at both the 3rd (*p* = 0.017) and 6th months post-op (*p* = 0.028) compared to the baseline level, while the reduction in RANKL level was significant only between the baseline and the 6th month post-op (*p* = 0.004). In the control group treated with conventional method, the RANKL level decreased significantly at each sampling time whereas sclerostin decreased significantly compared to the baseline level only at the 6th month after treatment (*p* = 0.001). OPG levels did not differ significantly in both groups throughout the entire follow-up period (Table 4).

**Table 4:** Comparisons of biochemical parameters during all study period

Biochemical Variables	Laser Group (*N* = 19) mean ± SD (min-max)				Control Group (*N* = 18) mean ± SD (min-max)				Laser Group vs. Control Group *p*^2^		
	Baseline	3 Months	6 Months	*p* ^1^	Baseline	3 Months	6 Months	*p* ^1^	Baseline	3 Months	6 Months
**IL-17 (ng/mL)**	16.26 ± 4.80 (6.90-24.53)	12.97 ± 5.07 ^a, c^ (5.11–24.13)	7.50 ± 5.03 ^b^ (1.66–18.26)	**< 0.0001** ^*^	19.74 ± 5.76 (7.92–29.25)	18.60 ± 8.29 ^d, f^ (2.03–32.48)	12.77 ± 8.22 ^e^ (1.61–27.94)	**< 0.0001** ^*^	0.245	**0.017** ^*^	0.057
**IL-10 (pg/mL)**	4.33 ± 2.79 (0.04–9.42)	2.35 ± 2.01 (0.03-6.00)	0.91 ± 1.05^b^ (0.03–3.19)	**< 0.0001** ^*^	7.45 ± 5.34 (0.47–17.56)	1.68 ± 1.79^f^ (0.29–6.16)	1.59 ± 1.84^e^ (0.27–5.37)	**< 0.0001** ^*^	0.053	0.538	0.245
**IL-1ß (pg/ml)**	10.08 ± 4.89 (5.02–23.93)	7.30 ± 3.89 ^a, c^ (2.36–17.94)	4.25 ± 2.23 ^b^ (0.95–8.74)	**< 0.0001** ^*^	14.92 ± 7.04 (6.47-28.387)	9.62 ± 4.61^d^ (5.03–19.92)	5.61 ± 2.21^e^ (0.52–9.97)	**< 0.0001** ^*^	0.19	**0.049** ^*^	0.07
**TWEAK (pg/ml)**	18.32 ± 4.67 (10-26.09)	12.89 ± 4.27 ^a, c^ (5.89-21)	5.33 ± 3.72 ^b^ (2.2-14.54)	**< 0.0001** ^*^	17.46 ± 6.92 (2.83–28.31)	11.66 ± 5.96 ^d, f^ (2.51–21.54)	7.48 ± 6.11^e^ (1.47–19.48)	**< 0.0001** ^*^	0.988	0.461	0.159
**Sclerostin (ng/ml)**	0.16 ± 0.07 (0.03–0.32)	0.11 ± 0.05^a^ (0.02–0.2)	0.11 ± 0.10 ^b^ (0.02–0.48)	**0.008** ^*^	0.24 ± 0.13 (0.11–0.52)	0.19 ± 0.13 (0.08–0.68)	0.13 ± 0.07 ^e^ (0.02–0.27)	**0.001** ^*^	0.070	**0.022** ^*^	0.150
**RANKL (pg/ml)**	1.09 ± 0.71 (0.09–2.25)	0.89 ± 0.60 (0.09–2.26)	0.66 ± 0.98 ^b^ (0.09–4.48)	**< 0.0001** ^*^	1.34 ± 0.53 (0.1–2.29)	1.09 ± 0.6 ^d, f^ (0.1–1.92)	0.55 ± 0.47 ^e^ (0.1–1.60)	**< 0.0001** ^*^	0.312	0.245	0.730
**OPG (ng/ml)**	0.025 ± 0.031 (0.004–0.027)	0.014 ± 0.009 (0.001–0.041)	0.018 ± 0.012 (0.0009–0.041)	0.069	0.023 ± 0.02 (0.001–0.07)	0.018 ± 0.014 (0.001–0.051)	0.016 ± 0.007 (0.002–0.026)	0.959	0.775	0.443	0.916

p^1^: Related Samples Friedman’s Two-Way Analysis of Variance by Ranks

p^2^: Mann Whitney U Test

^a^: Statistically significant difference between baseline and 3 months in the laser group

^b^: Statistically significant difference between baseline and 6 months in the laser group

^c^: Statistically significant difference between 3 months and 6 months in the laser group

^d^: Statistically significant difference between baseline and 3 months in the control group

^e^: Statistically significant difference between baseline and 6 months in the control group

^f^: Statistically significant difference between 3 months and 6 months in the control group

## Discussion

This study was designed to determine both clinically and biochemically if diode laser has any positive effect on the treatment of peri-implantitis. The test group received non-surgical mechanical debridement with titanium curettes and diode laser application; whereas the control group only received non-surgical mechanical debridement with titanium curettes. Assessing the clinical alterations in peri-implant tissues as well as the analysis of biomarker levels implicated in the pathogenesis of peri-implantitis both pre and post treatment interventions could enlighten the process of peri-implantitis development and also the ambiguity in its treatment. To our knowledge, this is the first study evaluating PICF IL-17, TWEAK and sclerostin levels after mechanical nonsurgical treatment combined with diode laser therapy in peri-implantitis.

Although several surgical and non-surgical treatment options have been recommended for the treatment of peri-implantitis [8, 32], there is no treatment protocol that can be considered as the gold standard yet. The efficacy of mechanical debridement alone is limited therefore, chemotherapeutics, anti-infective agents and lasers have been used as an adjunctive to mechanical treatment [33]. In the present study, adjunctive use of diode laser to non-surgical mechanical therapy was preferred due to its features such as bactericidal effect, coagulation, induction of fibroblast growth, the formation of a stable junctional epithelium and its ability to stimulate wound healing [34]. There are limited randomized controlled clinical studies in the literature on the use of diode laser in addition to non-surgical treatment in the treatment of peri-implantitis [20, 22, 24], and only one of these studies evaluated biomarkers in PICF [24].

In the present investigation, clinical periodontal parameters displayed significant decreases in both groups from the baseline to the 3rd month and from the baseline to the 6th month. Also, there was no significant difference between the effectiveness of the treatment groups in reducing pocket depth. These findings support that both treatment methods have similar efficiency in reducing pocket depth. However, the values of GI, GBTI, PI, except PD were lower in the laser group at the 3rd month follow-up. In line with our results, it has been reported that there is no difference in PD reduction between the groups with and without diode laser application [22, 24]. However, it has been demonstrated that there was a significant difference in PD alteration between peri-implantitis patients who received conventional mechanical treatment and the group who received diode laser along with conventional treatment [20] This difference may have occurred due to the fact that in the study by Lerario et al. peri-implant assessment was made based on the site rather than the dental implant, merely laser-treated patients were prescribed mouthwash containing chlorhexidine, and 4 out of 6 patients in the control group were smokers [20]. While GI and GBTI values differed in favor of laser group at the 3rd month, this difference vanished in the 6th month. This significant reduction in the early period following treatment was similar to the follow-up results of Arisan et al. at the first month [22]. It may be thought that the short-term impact of laser application is due to coagulation or vaporization in the soft tissue [35] but the lack of a marked difference between the groups at the 6th month can be interpreted as this advantage provided by diode laser just relating to the early healing period. In this study, the parallel course of PI changes with GI and GBTI alterations supported the relationship between plaque and gingival inflammation [36]. The decrease in PI observed over time in both groups has been similar with studies in the literature examining the effectiveness of diode laser [22, 24]. Although there are no studies evaluating the PICF volume in the studies investigating the effectiveness of diode laser, it has been reported that the PICF volume increases in the presence of peri-implantitis [25, 37]. In our study, the continuous reduction in PCIF during the follow-up periods in both groups can be assumed that the inflammation resolved over time.

Significant decreases in PICF IL-1ß, IL-17 and TWEAK levels were detected over time in both groups during follow-up periods. Supporting our results, there have been previous studies in which the pro-inflammatory cytokines IL-17 and IL-1β were detected higher in peri-implantitis compared to peri-implant health [32–40]. The significant decrease in IL-1ß and IL-17 PICF levels in the laser group compared to the controls at the 3rd month may be explained by the increase in early vascularization, acceleration of wound healing and bactericidal effect provided by diode laser application [41, 42]. However, it was observed that this improvement was only in the early period and there was no difference between the groups at 6th month follow-up. Yakar et al. [25] and Jansson et al.‘s [43] studies showing that TWEAK levels, a pro-inflammatory biomarker, in healthy peri-implant areas were significantly lower than in areas with peri-implantitis, have confirmed our outcomes.

The results of several studies comparing IL-10 levels in peri-implantitis and healthy peri-implant tissues have been contradictory [39, 40, 44]. Our findings are in line with the studies in which IL-10 levels were found higher in the presence of peri-implantitis than in healthy controls [40, 44]. It has been reported that there was an inverse relationship between IL-10 level and peri-implant PD [45] and this has been attributed to the fact that severe inflammation in deep pockets has changed the inflammation balance in favor of pro-inflammatory cytokines and against anti-inflammatory cytokines [39, 45]. In our study, the significant time-dependent decrease in PICF IL-10 levels after the treatments may be interpreted as the resolution of inflammation with peri-implantitis treatment and thus the decrease in anti-inflammatory as well as pro-inflammatory cytokines in the region [46]. However, the lack of a significant difference in IL-10 levels between the groups has suggested that laser application did not provide an additional benefit on reducing the levels of IL-10 in PICF.

The PICF values of sclerostin and RANKL, which are biomarkers associated with bone resorption, showed significant reduction following peri-implantitis treatments in both groups. Although there was no difference in sclerostin, which is up-regulated by the presence of pro-inflammatory cytokines, at 6th month, as in IL-17 and IL-1ß, the fact that these markers were observed significantly lower in the laser group at 3rd month can be explained by the early anti-inflammatory impacts of diode laser application [41]. The finding that RANKL in PICF decreased significantly as a result of peri-implantitis treatment in both groups in our study is compatible with studies in which this biomarker was higher in samples with peri-implantitis than in healthy samples [47, 48]. As peri-implantitis is characterized by progressive bone destruction, the reduction in RANKL level, which is involved in osteoclast activation [49], until the 6th month in both treatment groups may indicate a decrease in bone loss in the peri-implantitis area. No differences were observed within and between the groups in terms of OPG, which inhibits bone resorption by binding to RANKL. This outcome is in line with a recently published meta-analysis which stated that no statistically significant difference was detected in OPG levels when healthy and peri-implantitis areas were compared supported the findings of the present research.

Potential limitations of this study include the lack of a split-mouth design, conducting with a relatively limited number of samples, and the short follow-up period. Therefore, further studies with higher participation and longer follow-up periods are needed to understand the immunological changes in the peri-implantitis and to achieve strong evidence about the efficiency of laser application on clinical parameters and biomarkers in PICF.

## Conclusions

Within the limitations of the present study, significant improvements in clinical and biochemical parameters over time in the laser and control groups displayed that both treatment approaches were successful in the treatment of peri-implantitis with 6-month outcomes.

## Data Availability

The data that support the findings of this study are available from the corresponding author upon reasonable request. The data are not publicly available because of privacy or ethical restrictions.
